# Falls in older adults: a practical approach

**DOI:** 10.1590/0004-282X-ANP-2022-S107

**Published:** 2022-08-12

**Authors:** Cristiana Borges Pereira, Aline Mizuta Kozoroski Kanashiro

**Affiliations:** 1 Universidade de São Paulo, Faculdade de Medicina, Hospital das Clínicas, Departamento de Neurologia, São Paulo SP, Brazil.; 2 Hospital Regional de Mato Grosso do Sul , Departamento de Neurologia, Campo Grande MS, Brazil.

**Keywords:** Accidental Falls, Aged, Acidentes por Quedas, Idoso

## Abstract

**Background:**

Falls are a major problem in public health since they are an important cause of morbidity and mortality. To evaluate the risk of fall and prescribe preventive interventions may be a challenging task.

**Objectives:**

The objectives of this study are to summarize the most relevant information on the topic “falls in the elderly” and to give a critical view and practical clinical approach on this topic.

**Methods:**

In March 2022, a search of Pubmed database was performed, using the terms “fall elderly”, fall prevention”, “fall risk”, with the following parameters: five years, review, systematic review, meta-analysis, practice guidelines.

**Results:**

There are several risk factors for falls that can be grouped in different areas (psychosocial, demographic, medical, medication, behavioral, environmental). The clinical evaluation of an older adult prone to falls must include identification of risk factors through history and examination and identification of risk of falls through an assessment tool such as gait velocity, functional reach test, timed up and go, Berg balance test, and miniBEST test. Fall prevention strategies can be single or multiple, and physical activity is the most cited. Technology can be used to detect and prevent falls.

**Conclusion:**

A systematic approach to the older patient in risk of falls is feasible and may impact fall prevention.

## RESUMO

**Antecedentes:**

As quedas são um grande problema de saúde pública, uma vez que são uma importante causa de morbidade e mortalidade. A avaliação do risco de queda e a prescrição de intervenções preventivas podem ser tarefas desafiadoras.

**Objetivo:**

Estudo visa sintetizar as informações mais relevantes sobre o tema “quedas em idosos” e apresentar uma visão crítica e uma abordagem da prática clínica neste tema.

**Métodos:**

Em março de 2022, foi feita uma revisão bibliográfica no Pubmed, utilizando-se os termos “fall elder”, fall prevention”, “fall risk”, com os seguintes parâmetros s: 5 anos, revisão, revisão sistemática, meta-análise, diretrizes práticas.

**Resultados:**

Há inúmeros fatores de risco para quedas que podem ser agrupados em diferentes domínios (psicossocial, demográfico, condições clínicas, uso de medicamentos, comportamental, ambiental). A avaliação clínica de um idoso propenso a queda deve incluir a identificação de fatores de risco por meio da história e exame físico e identificação do risco de queda por meio de um instrumento de avaliação como velocidade da marcha, teste de alcance funcional, timed up and go, teste de equilíbrio de Berg, escala miniBEST. As estratégias de prevenção de quedas podem ser únicas ou múltiplas, sendo a atividade física a mais citada. Tecnologias podem ser usadas para detectar e prevenir quedas.

**Conclusão:**

Uma abordagem sistemática ao paciente idoso em risco de queda é possível, deve ser realizada e pode impactar na prevenção de quedas.

## INTRODUCTION

Falls are a major problem in public health since they are an important cause of morbidity and mortality. Fall rates vary among older adults according to age, but it is estimated that 30% of people over the age of 65 fall each year, and that this percentage increases up to 50% among people over 80^1^ ^-^ ^3^, while half of these have recurrent falls^4^. Added to this, falls lead to mild to severe injuries, are the cause of 10-15% of all emergency department visits, and account for 40% of all injury deaths^3^. Besides physical consequences, falls also can result in psychological sequelae characterized by fear of falling, insecurity, self-limitation, functional impairment, and social isolation. This situation compromises the quality of life and increases the risk of further falls^3^.

**Figure 1.**
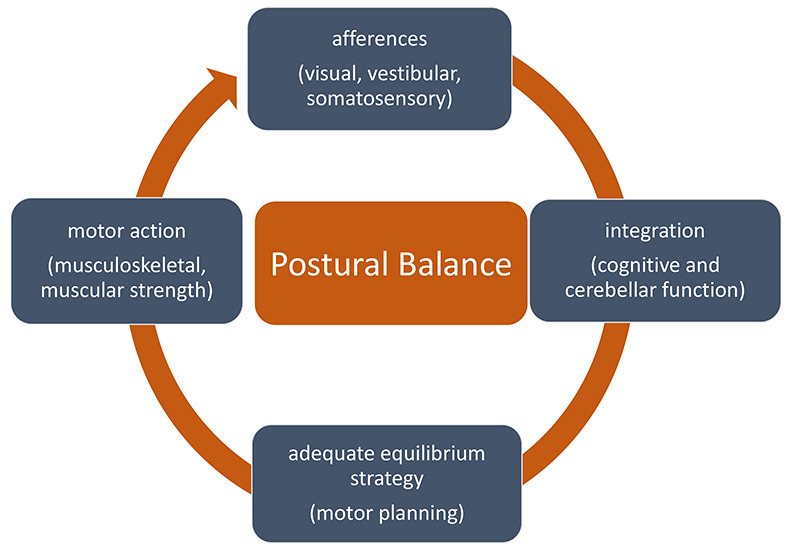
Schematic representation of all systems involved in postural balance.

The maintenance of a good postural balance is a complex task that involves an interaction to the environment, multiple afferences to the central nervous system, a complex integration, an efficient motor postural response and a good biomechanical support (Figure 1). Therefore, it is easy to understand that a fall can be the result of a problem in one or more of any of these stages. On the other hand, since there are several possible problems in this schema, identifying fall risks may be a challenging task. Furthermore, in most cases there are multiple risk factors involved and the fall cannot be attributed to a single cause. To evaluate the risk of fall of a patient may be another challenge, since there are different functional tools, with no consensus in the literature as to which one should be applied.

Thus, the aims of this review are to summarize the most relevant information on the topic “falls in the elderly” and to give a critical view and practical clinical approach in these cases. For this, the authors will answer the following questions: (1) Which are the most relevant fall risks in the elderly? (2) What is relevant in the physical and neurological examination? (3) How can one identify the risk of fall? (4) Which are the most important interventions to prevent falls? (5) How can new technologies help the management of older people with risk of fall?

## FALL RISKS ASSESSMENT

**Figure 2.**
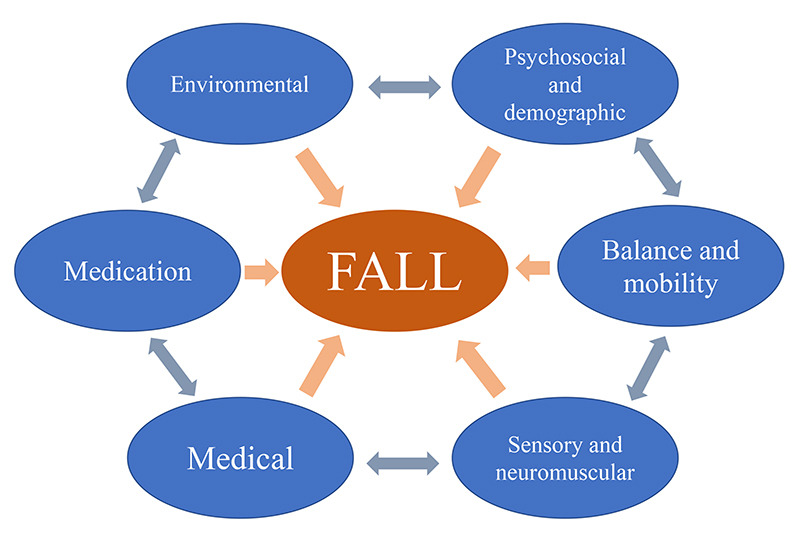
Schematic representation of fall risks domains. The arrows show the interaction between domains.

Publications describe an extensive list of risk factors for falls in the elderly, and there is a great effort to establish those that are most important, although the models are still insufficient to predict those patients at risk. The predictive accuracy of risk factor models for falls is weak compared with models for cardiovascular events, and one of the main risk factors for falls in the elderly is a previous fall, meaning there is no proper primary prevention as yet^4^. One of the possible explanations for this is that risk factors for falls are not static, they can interact with each other, adding to or multiplying their impact^4^ (Figure 2).

Therefore, screening the patient for risk factors for falls may be exhaustive. Epidemiological studies show dozens of risk factors, and some authors group these in different areas in order to make the analysis easier (Table 1) ^3^ ^,^ ^5^ ^,^ ^6^. 

**Table 1.** Fall risks factors grouped in different domains according to different authors.

WHO^3^	Lord et al^5^	Kim et al^6^
Biological risk factors	Psychosocial and demographic	General characteristics
Age Race Gender chronic illnesses (eg, neurologic diseases, arthritis, cancer) physical, sensory, cognitive, and affective declines	Advanced age Female gender Living alone History of falls Inadivity ADL limitations	Sex Increased age Living alone Low income
	Balance and mobility	Physical function
	Impaired stability when standing Impaired stability when leaning and reaching Inadequate responses to external perturbations Slow voluntary stepping Impaired gait and mobility Impaired ability in standing up Impaired ability with transfers	Low vision Mobility/balance/gait deficit Impaired ADL Musculoskeletal function Cardiac function Neurological function Inappropriate footwear Use of assistive devices
	Sensory and neuromuscular	
	Visual acuity Visual contrast sensitivity Visual field dependence Reduced peripheral sensation Reduced vestibular function Muscle weakness Poor reaction time	
	Medical	Disease factor
	Impaired cognition Depression Abnormal neurological signs Stroke Incontinence Acute illness Parkinson 's disease Vestibular disorders Arthritis Foot problems Dizziness	Stroke Dementia Parkinson’s Dizziness Cardiovascular Hypotension Respiratory Peripheral neuropathy Diabetes Chronic pain Arthritis Osteoporosis Incontinence
Behavioral risk factors		Behavior factor
Multiple medications lack of exercise excess alcohol usage inappropriate footwear inattention, multitasking hurrying		Inadequate diet History of previous falls Fear of falling Lack of exercise Excess alcohol intake
	Medication	Medication
	Psychoactive drugs Antihypertensive Use of four or more medications	Psychoactive drugs Cardiovascular drugs Multiple medication use
Environmental	Environmental	Environmental
narrow steps slippery surfaces of stairs looser rugs and insufficient lighting	Poor footwear Inappropriate spectacles	Brightness of light Carpet Slipperiness Support of community
Socioeconomic risk factors		
low income low education inadequate housing lack of social interaction limited access to health and social care lack of community resources		

However, a careful analysis shows that some factors are present in the three lists: age and sex, medication, medical conditions such as muscle weakness and visual changes, cognitive changes and depression, lack of physical activity/sedentary lifestyle. Next, we will comment on fall risks that we consider most relevant.

### General characteristics

Falls incidence increases with age, a finding easy to understand since many other factors (balance problems, polypharmacy, sensory and muscular impairment) are more common in older people. Women are more prone to falls than men, although fall-related mortality is higher among older men^3^. Biological difference may contribute to a higher risk in women, as they have a faster muscle mass sarcopenia than men. On the other hand, men may have a higher fatality rate, maybe because they only seek medical assistance when they have a severe condition, and they are more engaged in dangerous and risky activities and behaviors^3^. 

Falls among older people are associated with living alone^3^ ^,^ ^7^. A possible link between living alone and falls is depression, since isolation may increase depression, which increases the fear of falling, and vice-versa. Fear of falling can lead to less social interaction, loss of personal contact, and reduction of daily activity. This in turn reduces mobility and increases isolation and depression, and consequently increases the risk of falls^7^. 

### Medical conditions

Most authors agree that medical conditions such as stroke, dementia, Parkinson’s disease and peripheral neuropathy increase the risk of fall^5^ ^,^ ^6^. This is probably due to motor or cognitive dysfunctions that affect postural control. 

There is a close relationship between gait and cognition, and both can be impaired in early stages of neurodegenerative disorders. Cognitive impairment is associated with disturbed gait parameters, falls are more frequent in patients with dementia, and the number of falls increases with the severity of cognitive disturbance^8^.

Other conditions such as cardiovascular diseases may also increase the risk of falls, although it is not clear if this increase is due to disease itself, or due to the association with other conditions that occur with cognitive decline^6^. Regarding the association between falls and orthostatic hypotension, this may be difficult to confirm as it is an intermittent condition, and may not be present at the consultation but even so may be related to a fall^4^ ^,^ ^5^. Vestibular disease, dizziness, foot problems, and arthritis are also thought to be associated with an increased fall risk, but this has not been proven, probably due to a lack of evidence^5^ ^,^ ^9^. However, elderly patients with multisite pain had a 51% higher chance of fall risk^6^. Pain may cause low balance confidence, reduced self-protection from falls, mobility limitations and difficulties in activities of daily life and thus increases risk of fall^6^. 

### Balance, mobility, sensory, neuromuscular, and physical function

Considering the classification of Lord et al, medication and sensory and neuromuscular domains were associated with 50% greater risk for recurrent falls and balance and mobility and psychological domain with 30% greater risk^9^. 

Sensory and neuromuscular domain includes vision problems, peripheral sensation, and muscle weakness, so it is not a surprise that there exists an association with falls. 

Reduced visual acuity, contrast sensitivity, stereo-acuity and visual motion perception are all visual problems related to increased fall risk^10^. Presbyopia, cataracts, glaucoma, and macular degeneration are common causes of these visual problems. Furthermore, the use of bifocal and multifocal lenses worsens depth perception and sensitivity to contrast, increasing the risk of falling mainly when going up and down stairs outdoors^11^.

In elderly people with no neurological diseases, there may be a decrease of sensory afferences. The proprioceptive change occurs by axonal degeneration and a decrease in sensory fiber density and degeneration of chondrocytes on the cartilaginous surface due to articular degenerative disease. With a loss of proprioceptive sensation, the individual has greater difficulty when walking in environments with insufficient lighting, which increases the risk of falls^12^.

Elderly people lose 1 to 2% of muscle mass and strength each year. This process, called sarcopenia, is more pronounced in women after the age of 60 and is probably one of the reasons for a higher female incidence of falls^12^. 

Poor balance is one of the most important risk factors for falls, and the more difficult the balance task and the poorer performance of the task, the higher the risk of a fall^5^. Balance is also a complex ability and may also be linked to mobility and psychological aspects in the fall-risk assessment, as shown above. There is a corresponding relationship between falls, depression, mobility, and balance. The worse the balance and mobility, the higher the chance of depression and falls, while a fall increases the chance of depression, reduced mobility and poor balance^13^. 

Although supplementation of vitamin D may lower the risk of falls (see below), vitamin D deficiency is not included as a risk factor, since it is not a fall risk itself, but rather weakens the musculoskeletal system and thus may be associated with falls^6^.

### Medication

Polypharmacy (four or more drugs) or the use of specific classes of medication are important risk factors for falls^5^ ^,^ ^6^ ^,^ ^9^. Special care should be taken with psychoactive drugs (antidepressants, sedatives, opioids), anti-hypertensives (diuretics, b-blockers, vasodilators), skeletal muscle relaxants and antihistamine medication^14^ ^,^ ^15^. 

The particular situation of a patient prone to falls and with depression may be challenging, as depression and the use of antidepressants are both fall risks. In this case, if clinical conditions allow, the antidepressant should be withdrawn^16^.

### Behavioral and environmental factors

An important behavioral risk factor is the lack of physical activity. This is easy to understand, as physical activity is important for maintaining good health. It contributes to good muscular power and prevents functional decline^3^. 

Regarding environmental factors, although they are usually included in the list, there is some consensus that they do not cause falls by themselves, rather there is an interaction of external hazardous features and intrinsic risk factors^3^ ^,^ ^5^ ^,^ ^9^. In this context, the most common environmental factors cited are slippery surfaces, loose rugs, insufficient lighting, and inappropriate footwear. 

## PHYSICAL AND NEUROLOGICAL EXAMINATION AND ASSESSMENT TOOLS

The aims of a physical and neurological examination and the use of assessment tools are different, and the physician should bear this in mind. Through a physical and neurological examination, it is possible to identify the risk factors, and to evaluate the functional status of all systems involved with balance (musculoskeletal, muscle strength, coordination, sensibility, visual and vestibular function, cognition). On the other hand, by using one or more assessment tools the objective is to evaluate the mobility or balance function status and to identify the risk of fall. Thus, a complete evaluation should contemplate both aspects. 

### Physical and neurological examination

As mentioned above, a physical and neurological examination should focus on a physiological approach, rather than a disease-oriented approach^17^. As an example, the examiner must evaluate and identify possible visual problems such as poor visual acuity or visual contrast sensitivity rather than making a diagnosis of cataracts or glaucoma. With this in mind, all necessary systems for good balance and mobility must be examined: muscular strength and tonus, coordination, proprioception, visual function, vestibular function and cognition in special executive functions^14^ ^,^ ^17^. A simple evaluation of gait can also be helpful, observing velocity, stride length, antalgic movements and balance^14^. 

The examiner must also note that a marked deficit in one of these systems may alone explain a fall or disequilibrium. Nevertheless, most elderly patients have only mild to moderate deficits, and the combination of multiple impairments is in most cases the reason for falling^17^.

### Fall risk assessment tools

Once the patient has been examined and physical and neurological deficit has been identified, the physician can use one or two assessment tools to verify mobility, balance and the fall risk. 

Review papers about fall risk assessment list up to 28 different functional measurement tools^18^ ^,^ ^19^, and to make an analysis of all these tools is not our objective. Among all these tools, a few are often used and cited and are easy to perform in a practical and clinical approach and will be further discussed (Table 2). Although there is no consensus on which tests should be used, there is some agreement that an ideal evaluation should incorporate more than one test^18^ ^,^ ^20^.

**Table 2.** Assessment tools frequently used to identify risk of fall.

Single measure tools				
Test	Purpose	Instructions	Interpretation	Cut-off for falls
Functional reach test^21^	Measures the maximum distance a person can reach forward while standing in a fixed position	The patient is instructed to stand next to a wall and position the arm that is closer to the wall (but not touching) at 90 degrees of shoulder flexion with a closed fist. The assessor Instructs the patient to “Reach as far as you can forward without taking a step.” The location of the 3rd metacarpal is recorded at beginning and ending position, and the scores are the difference between these values. Three trials are done, final score is the average value of the best 2 results.	Smaller distances = worse performance	< 18,5cm^22^
Gait velocity^24^	Measures the walking speed in a comfortable, natural speed	A distance of 3-10 meters is measured, with or without an addition distance for acceleration and deceleration. Individual is timed once the foot touches the initial path and is stopped once it reached the end of the path (without the acceleration and deceleration distance). Two trials are measured	Higher gait velocity = better functional mobility Normal functional mobility = gait speed > 1,0m/sec	<0,7m/s^26^ <1,0 m/s^20^
Berg Balance Scale^27^	Assesses balance	14 items, that include static and dynamic balance tasks Each item points 0-4 Total scale score 0-56	Higher scores = better performance	45-49 points^29^
Timed up and Go (TUG)^30^	Assesses balance, mobility, walking ability	The patient sits on a standard armchair, and is instructed to stand up, walk on a comfortable speed along a l3 meters line, turn around at the line, walk back to the chair, and sit down. The test ends when the patient’s buttocks touch the seat.	Shorter time = better performance	>11 sec^20^ >13 sec^18^
miniBEST test^31^	Assesses balance, mobility, walking ability	14 items, that include dynamic balance tasks, walking tasks and TUG. Each item point 0-2 Total score 0-28	Higher scores = better performance	>19 points^33^ 17-25 points^32^ (age dependent)

These assessment tools can be divided into single-performance tests (gait velocity and functional reach test) and multidimensional performance tests (timed up and go, Berg balance test, miniBEST test). 

The objective functional reach test (FRT) is intended to evaluate how far the person can lean forward without taking a step or losing balance. Normal values are above 18,5cm ^21^ and values below that point are associated with an increased risk of fall^22^. 

Although walking may be seen as an easy task, it is quite a complex one and is considered a useful sign of functional mobility, as the slower the gait, the greater the chance of adverse events^23^. To measure gait velocity is very simple, but a few pitfalls must be mentioned: (1) the distance must be 3-10 meters, and longer distances give more reliable measures, (2) a small distance for acceleration and deceleration can be used, but the velocity must be measured only in a predetermined line; (3) the patient must be specifically instructed to walk at a comfortable, normal speed^24^. Normal gait speed is different for different ages, and older adults have a slower velocity^25^, thus cut-off values for risk of fall range from 0.7m/sec to 1.0m/sec^20^ ^,^ ^26^.

There is some controversy regarding the usefulness of the Berg balance scale^27^ for assessing fall risk in the elderly^28^ ^,^ ^29^, and a ceiling effect has been reported^28^, but this is still one of the most cited tools. A recent review and meta-analysis observed that cut-off for risk of fall between 45 and 49 points had a good predictive validity in sensitivity^29^.

The timed up and go test^30^ has long been used and a vast number of modifications such as walking as fast as possible, walking distances greater than three meters, additional cognitive or motor tasks have been described^18^. Normal values increase with age and range from eight seconds in people aged 60-69 to 12 seconds in those aged 80-89^25^. Thus, the cut-off for fall risk also shows variations from 11-13sec^18^ ^,^ ^20^. 

Through the mini-Balance Evaluation Systems Test (miniBESTest) scale it is possible to evaluate the performance in 14 tasks related to four aspects of dynamic balance: anticipatory adjustment, compensatory response, sensory organization, and gait. Each of the items can be graded between 0 and 2 points, the highest score being related to a better functional status^31^. Since older adults have a poorer performance and lower score, the cut-off values for fall risk in adults from 60 to above 90 range from 17 to 25 points^32^ ^,^ ^33^.

## INTERVENTIONS TO PREVENT FALLS

As falls in older people are multifactorial, relating to behavioral, environmental, socio-economic, and biological factors, efforts to prevent falls in the elderly should not be isolated. The prevention of falls in the elderly is supported by the Active Aging policy. According to the World Health Organization, Active Aging is “the process of optimizing opportunities for health, participation and security in order to enhance quality of life as people age”. The determinant factors of Active Aging are gender, culture, access to health and social services, behavioral determinants, physical environment, personal, social, and economic determinants^3^. Once functional capacity is impaired in the older age, fall prevention measures should be instituted to reduce falls and the complications resulting from them.

Based on the knowledge of risk factors for falls previously discussed, several studies have analyzed the effectiveness of simple and multiple interventions. Theoretically, for each fall risk factor there would be a prevention intervention. However, many risk factors are non-modifiable and prevention is limited. In this situation, promoting the understanding of these non-modifiable factors that result in falls can help to raise the awareness of older people and care providers that they should be more cautious in hazardous situations^5^. 

The interventions are often based on known modifiable risk factors for falling and many studies have evaluated the effectiveness of different interventions for the prevention of falls in the elderly. Many systematic reviews and meta-analysis studies have analyzed the effectiveness of different intervention programs to reduce the risk of falls, including single, multiple, and multifactorial interventions. Although studies have shown promising results, interventions have varied isolated or combined strategies resulting in data heterogeneity and difficulty in generalizing positive results^34^. 

Therefore, it has been necessary to develop a standardization through a taxonomy and common data collection system. Gillespie et al. categorized the interventions using the fall prevention classification system developed by the Prevention of Falls Network Europe (ProFaNe)^35^ ^,^ ^36^ (Table 3).

**Table 3.** Components of fall prevention.

Intervention	
Exercises (supervised, unsupervised, or both)	gait; balance and functional training; strength/resistance exercises; flexibility exercises; 3D training (e.g. Tai Chi); general physical activity; endurance training or others.
Medication (review and target)	vitamin D and calcium supplementation.
	medication withdrawal, dose reduction or increase, substitution or provision (antihypertensives, cardiovascular agents, drugs used in diabetes, anti-parkinson drugs, anti-dementia drugs, antidepressants, antipsychotic/neuroleptic drugs, anxiolytics/hypnotics/sedatives, other)
Surgery	cataract extraction, pacemaker provision, podiatric surgery or others.
Management of urinary incontinence	e.g. assisted toileting, bladder retraining, prompted voiding, pelvic floor exercises, antispasmodics
Fluid or nutrition therapy	Fluid therapy to restore the volume and composition of the body fluids to normal with respect to water- electrolyte balance. Nutrition therapy to improve health status of an individual by adjusting the quantities, qualities, and methods of nutrient intake.
Psychological individual or group	Cognitive (behavioural) interventions and others.
Environment/Assistive technology	Furnishings and adaptations to homes and other premises, aids for personal mobility, aids for communication, information and signalling, body worn aids for personal care and protection, social environment.
Social environment	staff ratio, staff training, service model, telephone support, caregiver training, home care services, others.
Knowledge	pamphlets, information, booklets/sheets, videos, lectures.

The interventions to prevent the fall risk in older people are classified into three major groups^37^:

- Single intervention;
- Multifactorial interventions: two or more interventions are given to a person according to the individual risk factor profile (e.g., the intervention exercise and home-hazard modification is applied to one person, whereas home-hazard modification and medication modification may be performed in another);
- Multiple interventions: two or more interventions are applied to all individuals participating in the fall prevention program (e.g., supervised exercise and vitamin D supplementation).

Thus, we will discuss the different prevention interventions to be performed alone or in combination (Table 4).

**Table 4.** Modifiable and non-modifiable fall risk factors and possible interventions to prevent falls.

Risk factor		Intervention
Non-modifiable conditions	Age Female gender	Raising awareness of the increased risk of falls
Medical conditions	Chronic diseases	Improving the individualized management of chronic diseases
	Depression	Improving the individualized management of depression Exercises
	Pain	Improving the individualized management of pain
	Impaired cognition	Improving the individualized management of impaired cognition Cognitive training, computerized version of cognitive training Exercises
	Visual impairment	Treatment with corrective lenses or surgery Exercises
	Balance impairment	Gait; balance and functional training; strength/resistance exercises; flexibility exercises; 3D training (e.g. Tai Chi); general physical activity; endurance training or others.
Medication	Polipharmacy	Medication review (withdrawal, dose reduction or increase, substitution, provision): anti-hypertensives, cardiac effects, hypoglycemic medications, skeletal muscle relaxants, antihistamine medications and mainly psychoactive drugs
	Supplementation	Vitamin D plus calcium
Behavioral	Living alone History of falls ADL limitations	Raising awareness of the increased risk of falls Possible change of living arrangements Aids for walking assistance: walking-sticks, walking-frames, wheelchairs
	Inactivity	Exercise, education
Environmental	Narrow steps	Advise for the use of appropriate footwear
	Slippery surfaces of stairs	Installation of safety features, correction or removal of hazards
	Looser rugs	Correction or removal
	Insufficient lighting	Correction of ambient lighting

### Interventions in non-modifiable conditions

For risk factors that have no scope for correction, such as advanced age and female gender, intervention strategies must be performed with the individual and caregivers raising awareness of the increased risk of falls^5^.

### Medical conditions

Many clinical and neurological conditions can contribute to the increased risk of falls. Intervention should be instituted as soon as the fall risk assessment is performed, regardless of a lack of evidence, in order to reduce the number of falls. In systematic review studies, for example, improving the management of chronic diseases, depression, and pain has been shown to potentially have much more effect on quality of life than on reducing falls, although this has not been tested^37^. 

Cognitive training (non-physical practice) can improve physical performance of older adults during single-task and dual-task walking (for example, walking while talking). It can be performed using a computerized version of cognitive training with a suggested session duration of 45 minutes or less. A shorter period of cognitive training helps to improve an acceptance of this intervention^38^. 

A vision assessment and treatment with corrective lenses or surgery, in combination with exercises, is strongly associated with reduction in injurious falls. The same is observed when multiple interventions are combined including exercises, vision assessment and treatment, environmental modification, and vitamin D supplementation^2^. 

### Exercises

Interventions with exercises are the most studied in prevention of falls and they have shown a reduction in both the rate of falls and the number of people who have falls. No difference was observed between groups of individuals with a high risk of falling or not. In all types of exercises the outcomes were better in reducing falls when interventions were performed by health professionals compared with interventions by trained instructors who were not health professionals^39^. The main categories of exercise programs associated with high-certainty evidence of fall reduction are balance and functional exercises. Interventions with multiple categories of exercises, mainly programs including balance and functional exercises plus resistance exercises, probably reduce falls (moderate-certainty evidence) though it was uncertain whether exercises classified as 3D (Tai Chi or similar) using the ProFaNE taxonomy reduced the rate of falls (very low-certainty evidence)^40^. Despite the latter interventions showing less evidence, the great heterogeneity observed in the systematic review studies does not allow a precise evaluation of these data.

Fall prevention interventions with exercise lasting less than six months were not effective in reducing the risk of falling. When these programs have a longer duration, lasting from six to 12 months or 12 months, the fall risk is reduced in 33% and 36%, respectively^41^. Similarly, the beneficial effects of different programs of exercises in fall risk in older people are related to the frequency of intervention. These effects were shown with a frequency of three to five times a week^42^. 

### Medication

Medication review is a necessary intervention that includes different actions (withdrawal, dose reduction or increase, substitution, provision) targeted to specific classes of drugs, mainly those that cause hypotension, cardiac effects, hypoglycemic medications, skeletal muscle relaxants, antihistamine medications and mainly drugs acting on the central nervous system^3^ ^,^ ^5^.

Regarding vitamin D supplementation, randomized controlled trials showed inconsistent findings, positive or negative, according to dosage of vitamin D^43^ ^,^ ^44^. However, the effectiveness of vitamin D supplementation for the prevention of falls is related to the basal serum levels of 25(OH)D in the elderly. Trials with vitamin D supplementation in older people with concentrations less than 20ng/mL demonstrated beneficial effects on preventing falls^45^. Unlike isolated vitamin D supplementation in fall prevention, the combination of vitamin D and calcium showed a 12% reduction in the risk of falls and benefits to musculoskeletal function and bone metabolism^45^ ^,^ ^46^. 

### Behavioral and environmental interventions

Fall prevention education promotes fall risk awareness and knowledge facilitating engagement in behavior and lifestyle changes, such as physical activity. Although the target of this intervention is the elderly, in order that it can attain positive results, it must be applied to both the elderly, caregivers, health professionals and the wider communities in which the older people live^3^.

Environmental interventions are related to the adaptations of homes, walking aids and aids for personal protection and changes in social environment mobility. Adaptation of homes refers for example to ambient lighting, suitability of beds, chairs, attention to rugs and slippery surfaces, and other factors. Walking-sticks, walking-frames, wheelchairs, orthopedic footwear are some aids for walking assistance and personal protection^3^.

## TECHNOLOGY IN FALL DETECTION AND PREVENTION

With advances in technology, researchers have developed techniques for detecting and preventing falls in the elderly, and some will be briefly discussed.

### Fall detection devices

Fall detection systems are used in order to establish whether an older person has had a fall through an alarm sent to health professionals, and so the consequences of falls are minimized. These systems continuously monitor older individuals using devices to find the fall prediction. These can be divided into three categories^47^ ^,^ ^48^:

- wearable devices consist of accelerometers, gyroscopes, magnetometers, etc. The posture and movement sensors process the information and decide if it is a fall or not. The decision is communicated to the pre-selected caregivers. The disadvantages of these devices are that they are intrusive and can be an extra burden to some individuals. Moreover, there is the risk of device displacement during everyday activities and less accurate results;
- camera-based devices, placed at selected locations around the individual for continuous monitoring. The major disadvantages of these systems are their limited coverage and that they compromise the user’s privacy;
- ambience devices that are a series of sensors strategically installed close to the individual, such as a wall, floor, bed, etc. The data are processed, and an algorithm decides whether there is a fall or not and then the caregivers are notified.

### Fall preventing devices

For the future, there are many machines learning algorithms still under development based on the data collected by sensors and data processing to identify and prevent falls.

Today a system of fall injury prevention is already available: the wearable airbag. This multisensory smart wearable belt detects collision with the ground surface. With a response time of 0.133 second it triggers two large-size airbags that lessen the fall impact. The limiting factors are the high cost and its effectiveness has not been evaluated on a large scale^49^. 

### Fall intervention devices

Fall intervention devices are used to improve the knowledge, mobility, and balance in older people. However, there is still no evidence that using these technology-based applications prevent falls^49^. The main examples of these technologies are: exergame and VR system; robot personal coaching system for fall education and walking-aid cane robot for real-time assessment of mobility and support to the user.

In conclusion, falls are a major problem in public health with physical and psychological consequences, compromising the quality of life and increasing the risk of further falls. 

Several risk factors for falls that can be grouped in different domains (psychosocial, demographic, medical, medication, behavioral, environmental) and the clinical evaluation of an older adult prone to falls must include identification of these risk factors through history and examination. Furthermore, identification of risk of fall must be done through an assessment tool such as gait velocity, functional reach test, timed up and go, Berg balance test, miniBEST test. Fall prevention strategies can be single or multiple, and physical activity is the most cited. Technologies can be used to detect and prevent falls. Thus, a systematic approach to the older patient at risk of fall is feasible and may impact fall prevention.
